# Non-perforated peptic ulcer disease: multidetector CT findings, complications, and differential diagnosis

**DOI:** 10.1007/s13244-017-0562-5

**Published:** 2017-07-04

**Authors:** Massimo Tonolini, Anna Maria Ierardi, Elena Bracchi, Paolo Magistrelli, Adriana Vella, Gianpaolo Carrafiello

**Affiliations:** 1 Department of Radiology, “Luigi Sacco” University Hospital, Via G.B. Grassi 74, 20157 Milan, Italy; 2 Diagnostic and Interventional Radiology Department, ASST Santi Paolo e Carlo, Via A di Rudinì 8, 20142 Milan, Italy

**Keywords:** Peptic disease, Gastroduodenal ulcer, Digestive haemorrhage, Gastric outlet obstruction, Computed tomography (CT)

## Abstract

**Abstract:**

Despite availability of effective therapies, peptic ulcer disease (PUD) remains a major global disease, resulting from a combination of persistent *Helicobacter pylori* infection and widespread use of nonsteroidal anti-inflammatory drugs. Albeit endoscopy definitely represents the mainstay diagnostic technique, patients presenting to emergency departments with unexplained abdominal pain generally undergo multidetector CT as an initial investigation. Although superficial ulcers generally remain inconspicuous, careful multiplanar CT interpretation may allow to detect deep ulcers, secondary mural and extraluminal signs of peptic gastroduodenitis, thereby allowing timely endoscopic verification and appropriate treatment. This pictorial essay aims to provide radiologists with an increased familiarity with CT diagnosis of non-perforated PUD, with emphasis on differential diagnosis. Following an overview of current disease epidemiology and complications, it explains the appropriate CT acquisition and interpretation techniques, and reviews with several examples the cross-sectional findings of uncomplicated PUD. Afterwards, the CT features of PUD complications such as ulcer haemorrhage, gastric outlet obstruction, biliary and pancreatic fistulisation are presented.

***Teaching points*:**

• *Gastric and duodenal peptic ulcers are increasingly caused by nonsteroidal anti-inflammatory drugs*

• *Multiplanar CT interpretation allows detecting deep ulcers and secondary signs of gastroduodenitis*

• *CT diagnosis of uncomplicated peptic disease relies on direct and indirect signs*

• *Currently the commonest complication, haemorrhage may be treated with transarterial embolisation*

• *Other uncommon complications include gastric outlet obstruction and biliopancreatic fistulisation*

## Introduction

Peptic ulcer disease (PUD) refers to development of gastric or duodenal mucosal defects which penetrate through the muscularis mucosa. Until the end of the twentieth century, PUD represented a major cause of morbidity and mortality worldwide. Since then, the availability of effective therapies, including H2-receptor antagonists, proton pump inhibitors and *Helicobacter pylori* (HP) eradication treatment, resulted in decreasing incidence of PUD. However, PUD continues to be a major global health issue that affects roughly 10% of the world population, due to the combined effect of persistent HP epidemics in low-income countries and of widespread use of nonsteroidal anti-inflammatory drugs (NSAIDs) [1, 2].

Patients affected by PUD may suffer from chronic symptoms such as dyspepsia, epigastric discomfort, nausea and early satiety, and often experience acute bouts of abdominal pain. Upper digestive endoscopy is definitely the mainstay diagnostic technique for PUD, but is invasive and often unfeasible in urgent conditions unless gastrointestinal bleeding is suspected. As a result, many patients with unknown PUD present to emergency departments with unexplained acute abdomen and commonly undergo multidetector CT. Since perforated gastroduodenal ulcers represent the commonest cause of spontaneous pneumoperitoneum, suggesting the underlying presence of PUD is relatively straightforward when radiologists are faced with upper abdominal free air or peritonitis without CT signs of colonic diverticulitis and digestive tract masses [3, 4].

Conversely, due to the widespread belief that CT has no role in detecting uncomplicated PUD, most non-perforated ulcers are missed at primary CT interpretation. Unfortunately, unless clinical suspicion of PUD exists, endoscopy may not be performed for days or weeks after CT, thus allowing time for complications to develop. Albeit superficial ulcers are generally inconspicuous, careful multiplanar CT interpretation and attention to subtle mural and extraluminal signs may allow diagnosing non-perforated PUD prospectively. Suggesting the possibility of unexpected PUD may allow planning early endoscopy for diagnostic confirmation and timely start of appropriate treatment with nasogastric tube aspiration, intravenous fluids, and HP “triple therapy” eradication [5–7].

Therefore, this pictorial essay aims to provide radiologists with an increased familiarity with CT appearances of non-perforated PUD. After an overview of the current epidemiology of PUD, we explain the appropriate multidetector CT acquisition and interpretation techniques. Then, the imaging findings of uncomplicated peptic disease, PUD-related digestive haemorrhage, gastric outlet obstruction and biliopancreatic fistulisation are presented with examples and a focus on their respective differential diagnoses.

## Epidemiology, pathogenesis and complications of peptic ulcer disease

Despite progressive eradication, over half of the world’s population still harbours chronic HP infection, particularly in developing countries. Conversely the prevalence of PUD is lower (approximately 1.5% to 5%) in Europe and North America, where an increasing proportion (up to 46% of patients) of PUD cases is secondary to the widespread use of NSAIDs including low-dose aspirin. Since NSAIDs and HP result in additive risk, in Western countries PUD is increasingly encountered in elderly people (with a 1.5 male predominance), who usually suffer from lower degrees of abdominal pain compared to their younger counterparts. Other factors such as cigarette smoking and alcohol intake may contribute to the risk of developing PUD. In 10% of cases the disease is idiopathic and unrelated to either HP or NSAID medications. Furthermore, PUD probably plays a role also in the pathogenesis of atrophic gastritis, gastric adenocarcinoma and mucosa-associated lymphatic tissue (MALT) lymphoma. Nowadays, albeit the more significant decrease has been in the duodenum, duodenal ulcers remain more common than their gastric counterparts [1, 2].

Currently, digestive bleeding is by far the commonest complication, followed by perforation, gastric outlet obstruction and fistulisation, in descending order of frequency. Risk factors associated with development of PUD complications include male sex, advanced age, comorbidities, alcohol, smoking, NSAID use, anticoagulation, corticosteroids and immunosuppressant medications. Mortality increases with age and comorbidities, and is higher in patients without previous history of PUD [1, 2, 8].

## Multidetector CT technique and interpretation

In the emergency setting, CT is largely used to rapidly assess patients with acute abdominal complaints, aiming to identify complications requiring hospitalization and surgery. In our experience, clinical suspicion of PUD is exceptional in urgent CT requests: as a result the disease most usually represents an unexpected finding in CT studies performed using routine abdominal protocols including a preliminary precontrast, an optional pancreatic phase (using a 35 s delay) and a mandatory portal-venous phase enhanced acquisition obtained 70–75 s after automated power injection of 110 to 130 ml of non-ionic iodinated contrast medium at a 2.5–3 ml/s flow rate, followed by saline flushing. When gastrointestinal bleeding is suspected, an arterial-dominant acquisition is performed using faster contrast injection (3.5–5 ml/s), bolus tracking with region-of-interest placed in the upper abdominal aorta and maximum-intensity projection (MIP) reconstructions; this technique is beneficial for identifying active contrast extravasation, which mandates endoscopic or interventional treatment. On our 64-slice CT scanner, the usual acquisition parameters used for abdominal studies are 120 KV, 300 mAs, 0.891 pitch, 0.75 s rotation time, and 64 × 0.625 mm collimation.

In most routine contrast-enhanced CT studies, the decompressed stomach and duodenum with peristalsis or retained enteric content are inherently difficult to assess. The inadequately distended stomach shows prominence of the gastric folds and apparent mural thickening; in absence of disease, the gastric wall thickness may reach up to 12 mm in the antrum. In patients with clinical suspicion or endoscopic detection of gastro-duodenal disease, the attending radiologist may opt for a dedicated CT study with gastric distension to assess the true mural thickness and structure. After fasting for at least 6 h, the patient is instructed to drink 500 to 750 ml of tap water as negative oral contrast during 15–20 min before examination and is then placed on the scanner table in either supine or prone (when antropyloric abnormalities are suspected) position. After hypotonisation using 20 mg of N-butylscopolamine intravenously, contrast-enhanced CT is obtained in pancreatic-dominant and portal venous phases [5–7, 9].

Multidetector CT studies are routinely reconstructed along axial and coronal planes. Furthermore, as seen in the following imaging examples, thorough search for gastric and duodenal abnormalities benefits from additional sagittal and oblique study review, particularly focused on elucidating anatomy and abnormalities of the antropyloric tract and proximal duodenum.

## CT appearances of uncomplicated peptic disease

**Fig. 1:**
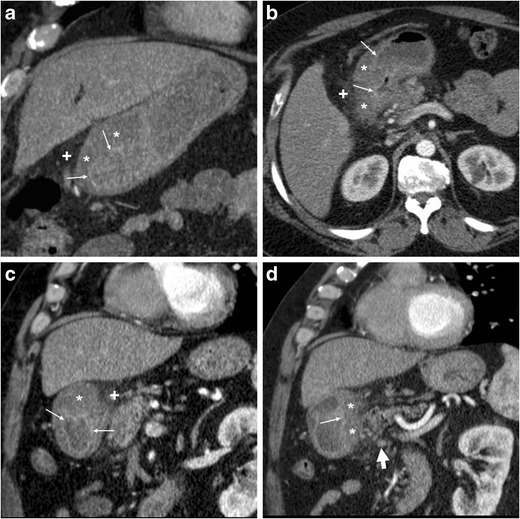
A 54-year-old obese male smoker, admitted to emergency department (ED) because of epigastric pain radiating to the back with deep abdominal tenderness, underwent urgent multidetector CT-angiography “to rule out acute aortic disease”. Secondary CT interpretation with multiplanar reconstructions identified findings consistent with peptic gastro-duodenitis, namely asymmetric oedematous mural thickening (*) with mucosal hyperenhancement (thin arrows) along the posterior and superior aspects of the distal antrum, pylorus and duodenal bulb, subtle perivisceral inflammatory stranding (+) and at least a centimetric lymphadenopathy (thick arrow in **d**) [Adapted from Open Access ref. no [10]]

**Fig. 2:**
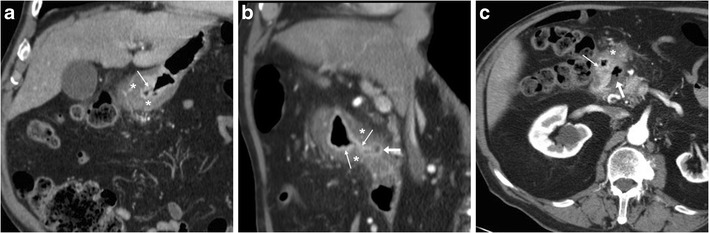
An elderly 87-year-old male underwent emergency contrast-enhanced CT to investigate acute abdominal pain with epigastric tenderness but no peritonism: the antropyloric region showed pronounced, stratified mural thickening with oedematous submucosa (*), mucosal hyperenhancement best seen in arterial-phase images (thin arrows), and a posterior prepyloric deep ulcer crater (arrows) filled by fluid and air, which was subsequently confirmed by endoscopy

**Fig. 3:**
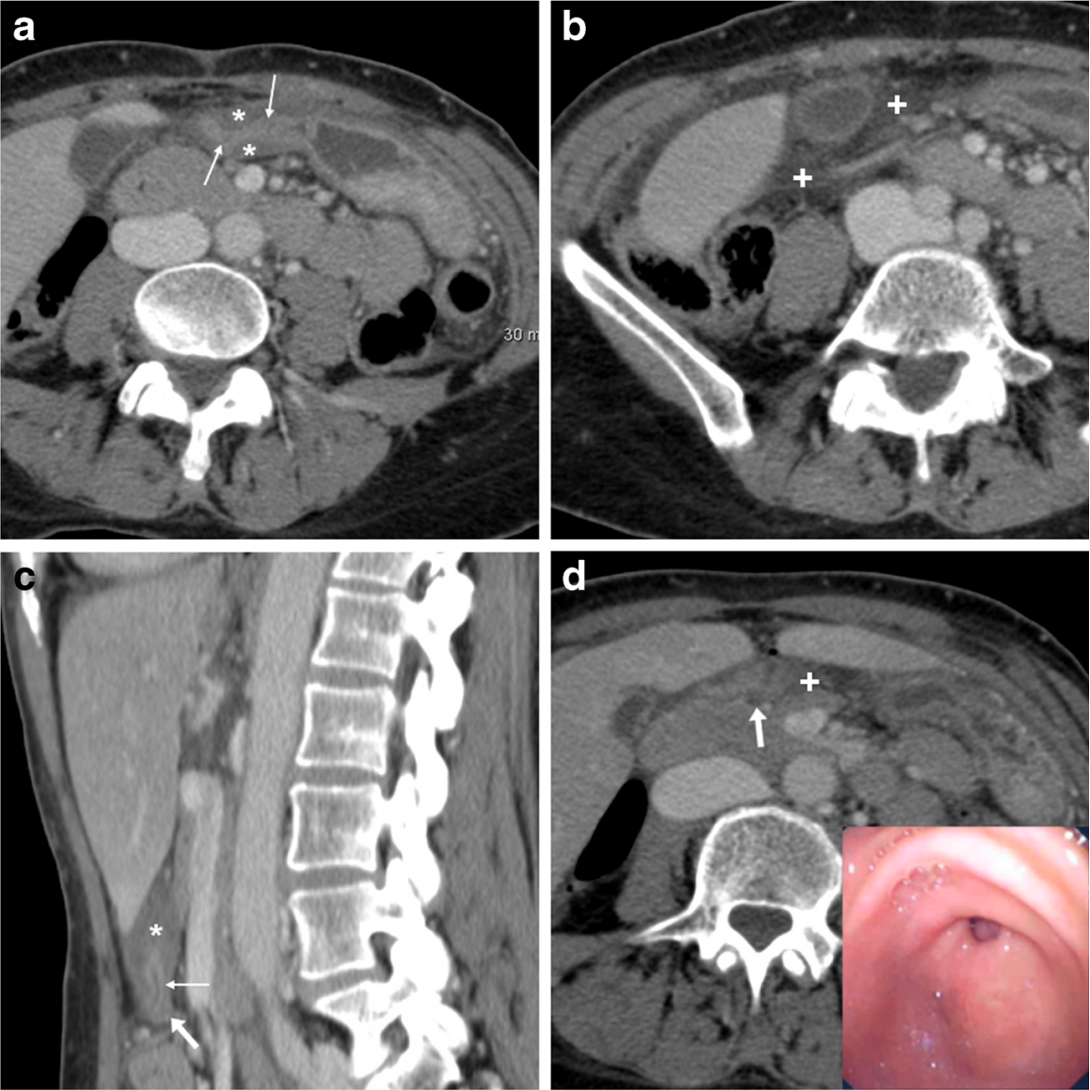
A 55-year-old female with unremarkable medical history experienced severe abdominal pain with left-sided tenderness and minimally elevated serum lipase. Multiplanar CT images showed collapsed gastric antrum with moderately thickened, stratified wall (*) and enhancing mucosa (thin arrows). Additionally, inflammatory hyperattenuation of surrounding and infrahepatic fat planes (+) reinforced suspicion of acute PUD. The subtle, small-sized mucosal defect with centimetric outpouching (arrows in **c**, **d**) corresponded to endoscopic finding (image in inset **d**) of posterior prepyloric ulcer with oedematous margins and fibrinous crater. The patient received medical therapy with continuous PPI infusion

**Fig. 4:**
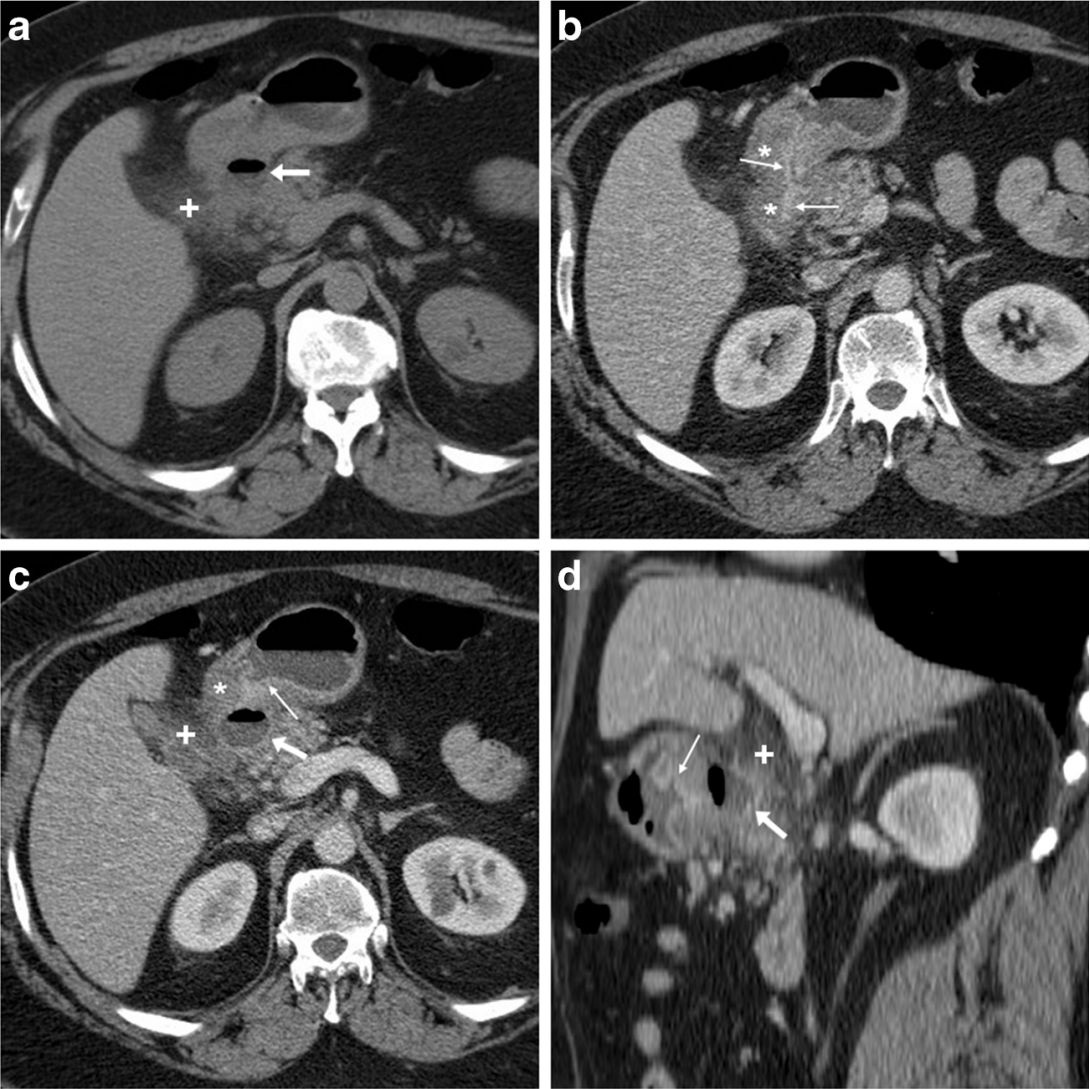
Three months later, the same patient in Fig. 1 experienced severe, recurrent abdominal pain with laboratory evidence of systemic inflammation (14.000/mmc leukocytes, 34 mg/L C-reactive protein). On further questioning, he admitted discontinuation of antacids and H2-blocker medications, and nonsteroidal anti-inflammatory drugs (NSAIDs) intake. Repeated CT revealed increased hypoattenuating mural thickening (*) with mucosal enhancement (thin arrows) of the pylorus and proximal duodenum which spared the anterior aspect, worsened periduodenal inflammatory changes (+), and development of a 2-cm roundish posterior deep ulcer (arrows). Upper digestive endoscopy confirmed a non-malignant retropyloric peptic ulcer. The patient ultimately did well on proton-pump inhibitors (PPI) and anti-*Helicobacter pylori* triple therapy [Adapted from Open Access ref. no [10]]

The CT diagnosis of PUD relies on a combination of direct and indirect signs. Among the latter, the hallmark of gastro-duodenitis is represented by mural thickening of the affected portion of the stomach and of the proximal duodenum, which typically shows a stratified appearance corresponding to hypoattenuating oedematous submucosa and enhancing hyperaemic mucosa. This appearance may be either diffuse or focal surrounding the ulceration site, and is best perceptible in views perpendicular to the long axis, and in arterial-dominant or pancreatic phases rather than in portal-venous acquisitions. In the setting of HP- or NSAID-related PUD, gastro-duodenitis is generally segmental and limited to the distal gastric body and antrum (Figs. 1, 2, 3 and 4). The other useful indirect sign which alerts the keen radiologist to the possible presence of an active inflammation is represented by oedematous “stranding” of the perigastric or periduodenal fat (Figs. 1, 3 and 4). Sometimes, adjacent adenopathies may be present (Fig. 1) [5–7].

The two direct signs of PUD include focal discontinuity of the mucosal hyperenhancement, which reflects disease reaching the muscularis mucosa, and identification of luminal outpouching: the latter corresponds to the ulcer crater which extends through and beyond the gastroduodenal wall (Figs. 2, 3 and 4). Literature from the multidetector CT era reported sensitivities of CT for ulcers of 29.6% to 54%, with an average size of 25 mm for visible ulcers compared to 16 mm of missed ones. Whereas even large ulcers may not be visible on CT with poor luminal distension, deep or penetrating ulcers may be identified with careful multiplanar scrutiny and paying attention to secondary findings [5–7].

### Differential diagnosis of uncomplicated peptic disease

**Fig. 5:**
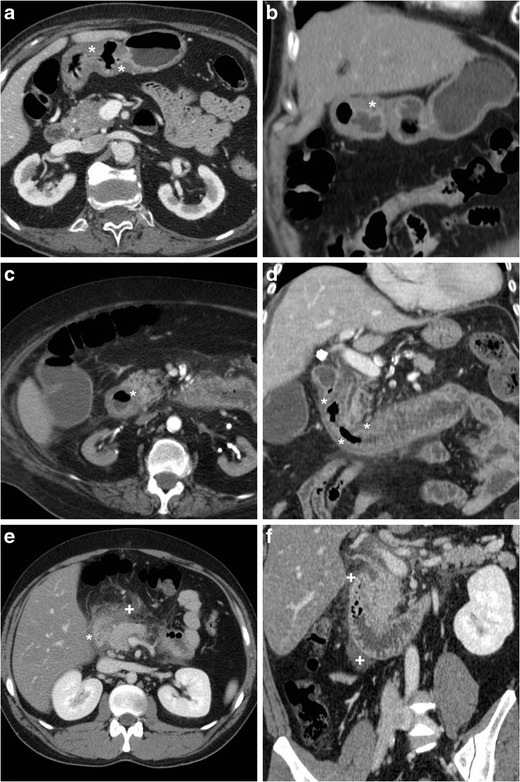
In a 34-year-old female with unspecific abdominal pain and unremarkable endoscopic findings, the contracted gastric antrum showed at CT (**a, b**) apparent hypoattenuating mural thickening (*), which mimicked characteristic appearance of acute peptic disease but interrupted at the site of spasms and lacked mucosal hyperenhancement. Non-inflammatory circumferential oedematous mural thickening (*) of the proximal and descending duodenum is seen in a 43-year-old female with anasarca due to renal failure (**c, d**). In a 29-year-old alcohol-addicted male with interstitial oedematous acute pancreatitis, contrast-enhanced CT (**e, f**) showed normally enhancing pancreas, ill-defined oedematous duodenal wall (*) with associated periduodenal fluid and fat stranding (+) extending from the proximal to the third duodenum

Caution should be paid to not overcall diagnosis of peptic gastro-duodenitis: in a contracted stomach, an apparent hypoattenuating mural thickening (Fig. 5a, b) may mimic the characteristic findings of acute PUD but interrupts at the site of spasms. Similar appearances may result from non-inflammatory oedema, such as in patients with liver failure or anasarca (Fig. 5c, d); however these conditions generally lack the mucosal hyperenhancement. The commonest form of duodenal inflammation is secondary involvement from acute pancreatitis ( Fig. 5d, e), which usually affects the descending duodenum along its medial aspect, rather than the duodenal bulb. Periduodenal inflammatory changes ranging from mild “hazy” stranding to mass-forming infiltration of the pancreatic-duodenal groove are characteristic of paraduodenal pancreatitis [11–14].

**Fig. 6:**
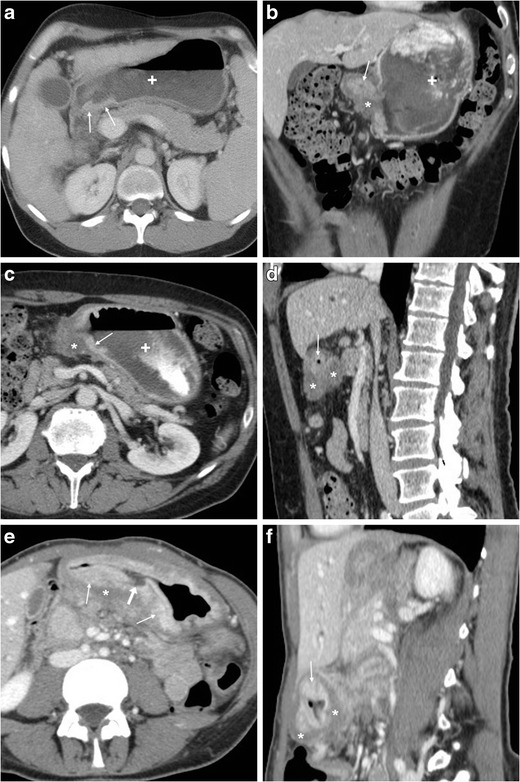
Differentiation of gastric tumours from PUD. In a 45-year-old HIV-positive male with wasting syndrome, CT (**a**) with oral water (+) showed localized, enhancing mural thickening at distal gastric antrum (thin arrows). In a 67-year-old male, CT (**b…d**) revealed distended gastric fundus (+) with residual contrast from previous fluoroscopic study, contracted antrum with mucosal enhancement (thin arrows) and hypoattenuating mural thickening (*); multiplanar image review revealed shouldering at transition between abnormal wall and upstream gastric body (in **c**) and eccentric, nodular-shaped thickening (in **d**) with subtle infiltration of perigastric fat, consistent with endoscopic diagnosis of stomach cancer. In a 44-year-old female, diffuse signet-ring gastric carcinoma (linitis plastica) showed CT (**e, f**) appearance of asymmetrically thickened, hypoattenuating wall (*) with irregular, enhancing mucosal thickening (thin arrows) and a focal posterior ulceration (arrow in **e**)

Particularly in the elderly, the key differential diagnosis of gastric or duodenal mural thickening is malignant tumours. Neoplastic mural thickening is typically focal, nodular or mass-forming rather than diffuse, often measures over 1 cm, shows uniformly soft-tissue attenuation rather than layered enhancement pattern (Fig. 6), and may infiltrate the surrounding fat. Albeit deep ulcerations which protrude into a solid mass strongly suggest cancer, to overlapping CT appearances (Fig. 6e, f) endoscopy is generally required for diagnostic confirmation [5–7].

**Fig. 7:**
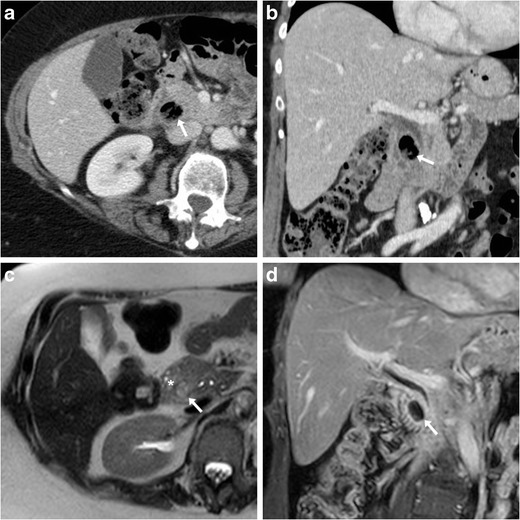
In a 77-year-old female with recurrent upper abdominal pain, axial (**a**) and coronal (**b**) post-contrast CT images showed a duodenal diverticulum (arrows) filled by air and enteral material, in typical medial site at distal descending duodenum. At MRI, T2-weighted (**c**) and fat-suppressed gadolinium-enhanced T1-weighted (**d**) images respectively showed oedematous, inflamed surrounding pancreatic parenchyma (+) and hyperenhancing contour of diverticulum (arrows)

Finally, ulcer outpouchings should be differentiated from duodenal diverticula, which are not uncommon in the elderly population, often asymptomatic, and typically located medially, at the transition between descending and horizontal portions (Fig. 7) [11–14].

## Peptic ulcer haemorrhage

Bleeding is nowadays the most common PUD complication, which affects up to 15% of patients (incidence 19–57 cases/100.000 individuals/year) with NSAID use as key risk factor. Manifestations range from asymptomatic blood loss with positive faecal blood test, to melaena or haematemesis, to hypovolemic shock. Despite effective treatment, PUD-related haemorrhage tends to recur and up to 31% of patients will experience rebleeding within a month [1, 15, 16].

**Fig. 8:**
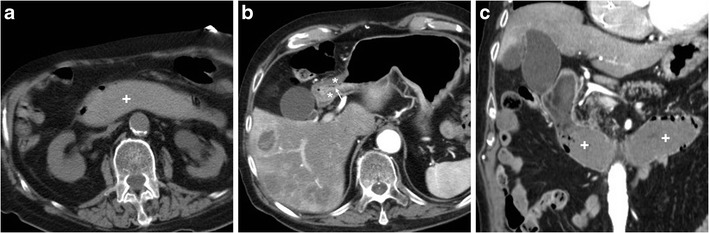
In an elderly 84-year-old male with haematemesis and melaena, precontrast CT scans (**a**) showed dilated second, third and fourth duodenum filled by hyper attenuating fluid (+) consistent with recent haemorrhage. Arterial-phase CT acquisition (**b, c**) showed characteristic appearance of antropyloric PUD with submucosal oedema (*) and mucosal hyperenhancement (thin arrow) but did not reveal signs of active bleeding, as confirmed by endoscopy

Albeit endoscopy remains the standard of care, as it allows both diagnosis and haemostasis, multidetector CT may prove useful in patients with suspected gastroduodenal bleeding. On preliminary unenhanced scans, dependent hyper attenuating debris from recent blood may be detected in the gastric fundus or duodenal lumen (Fig. 8). Blood products measure 30 to 45 Hounsfield units (HU), and the highest attenuation clot (45–70 HU) is generally found nearby the bleeding site. Caution should be paid to not misinterpret ingested material, medications, foreign bodies or residual contrast medium as blood, leading to falsely positive diagnosis.

**Fig. 9:**
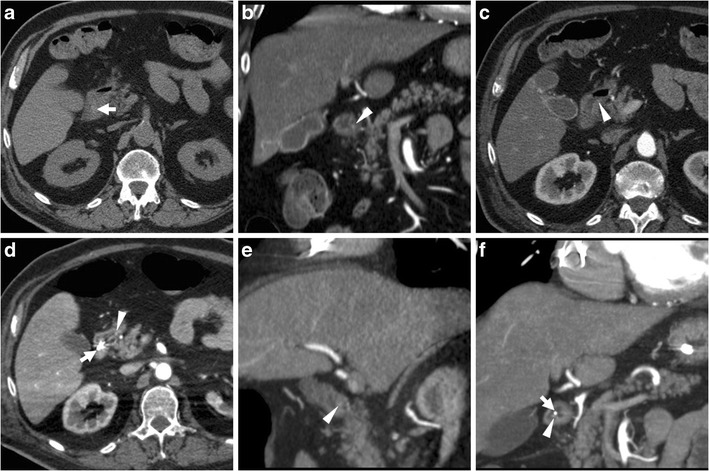
A 59-year-old female underwent emergency CT (**a…c**) because of hypotension and blood loss. Unenhanced scans (**a**) revealed subtle hyper attenuating content of proximal duodenum (thick arrows). Arterial-phase images (**b, c**) showed a dot-like mucosal enhancement (arrowheads) at dorsal aspect of duodenal bulb, which corresponded to bleeding ulcer and required endoscopic clipping and transfusions. Four days later, recurrent bleeding with melaena occurred: repeated CT-angiography (**d…f**) showed metallic clip (thick arrows) and thin jet-like intraluminal contrast extravasation (arrowheads), which was immediately treated with local epinephrine injection

**Fig. 10:**
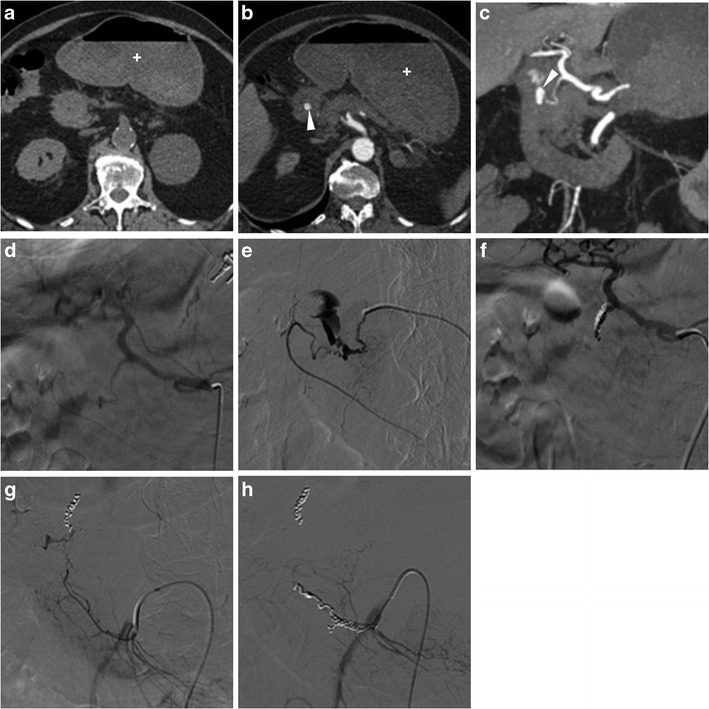
A 63-year-old, haemodynamically unstable (blood pressure 70/40 mmHg) female with massive haemoptysis (haemoglobin 5 g/dl) underwent CT-angiography following inconclusive endoscopy. Precontrast images (**a**) showed bloody fluid (+) in the stomach. Arterial-phase images (**a**) and Maximum-intensity projection (MIP, **b**) reconstructions revealed a focal contrast “blush” extravasation within the duodenal lumen (arrowheads) originating from the gastroduodenal artery (GDA). Angiography (**d**) and selective angiography (**e**) confirmed active bleeding from the GDA. Despite coil embolisation of the GDA (**f**) having obtained apparent exclusion of haemorrhage, angiography of the superior mesenteric artery (**g**) showed refilled bleeding through a branch of the arc of Buhler. Finally, after repeated embolisation on that second vessel, angiography (**h**) showed complete resolution of haemorrhage

With help from focused MIP reformations, multidetector CT may allow direct identification of active haemorrhage as intraluminal “jet-like” or “blush” contrast extravasation at the bleeding site, isoattenuating with enhanced vessels (Figs. 9, and 10). In PUD, arterial bleeding may originate from the gastroduodenal artery, such as in duodenal ulcers, or from the left gastric artery in gastric ulcers along the lesser curvature, and in our experience is generally focal and subtle. Alternatively, CT may suggest different causes of digestive haemorrhage, such as tumour or varices [5–7].

When endoscopic haemostasis cannot be achieved, transcatheter arterial embolisation (TAE) (Fig. 10) may be helpful, particularly in frail patients. Compared to surgery, TAE has a slightly lower rate of haemostasis (91% versus 100%) and more frequent rebleeding (40% versus 15%), but is associated with fewer complications and better overall outcome [17].

## Gastric outlet obstruction

Gastric outlet obstruction (GOO) refers to blocked passage of gastric contents into the duodenum from a partial or complete obstacle located at the distal stomach, pylorus, or duodenal bulb. Manifestations generally include vomiting and inability to eat. Currently, obstruction represents an uncommon PUD complication with a 2% incidence, in 80% of cases secondary to scarring from prolonged inflammation in untreated or long-standing bulbar ulcers [1, 15].

**Fig. 11:**
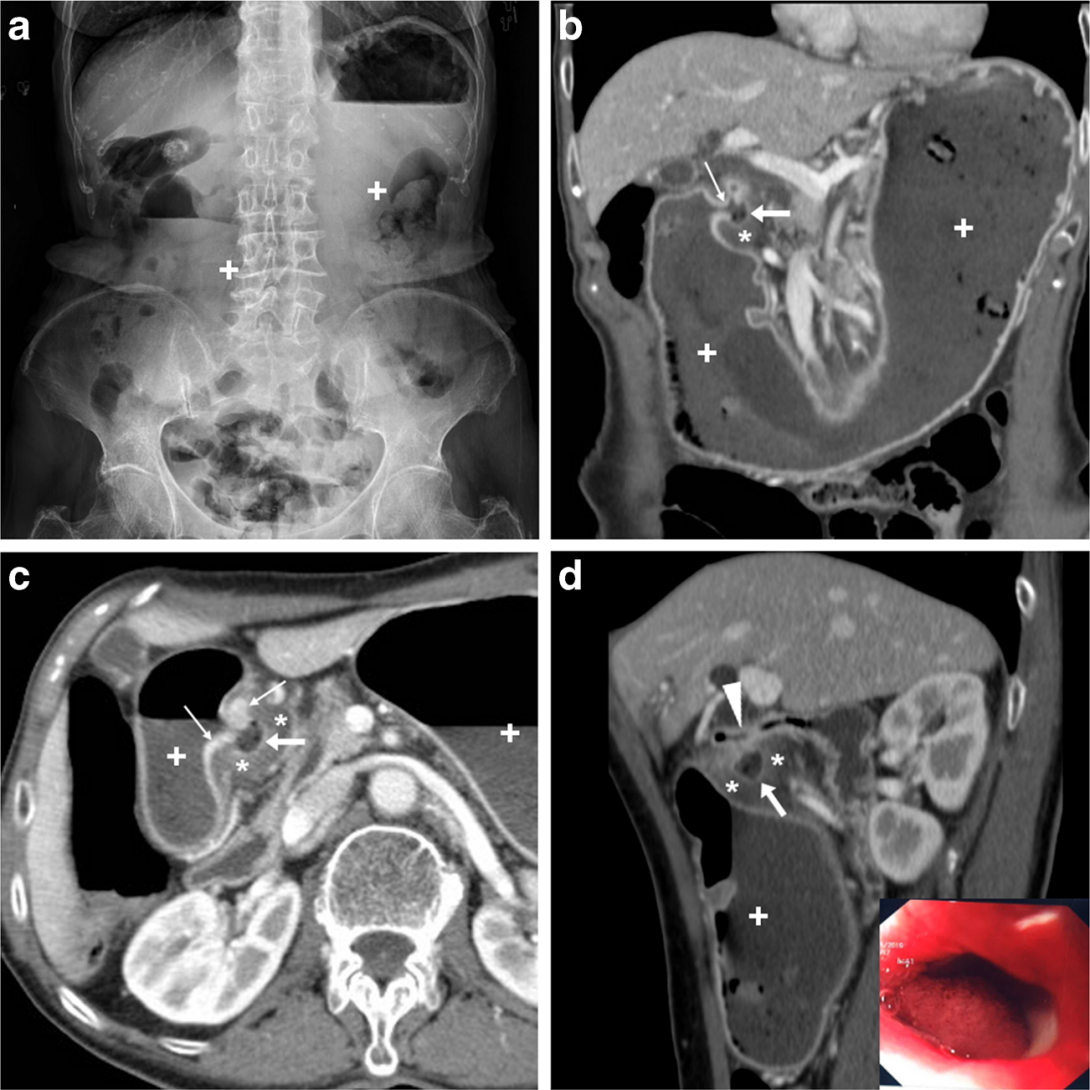
In a 68-year-old woman suffering from recurrent abdominal pain, nausea and 10-kg weight loss, abdominal radiographs (**a**) showed gastric overdistension by intraluminal stagnant fluid (+) despite fasting. Contrast-enhanced multidetector CT (**b…d**) confirmed overdistended stomach with normal mural thickness. The contracted pylorus and proximal duodenum showed circumferential hypoenhancing mural thickening (*) with mucosal hyperenhancement (thin arrows) focally interrupted at the site of an ulcer crater (arrows). Endoscopy confirmed moderate stricture of the proximal duodenum and post-pyloric ulcer (image in inset **d**) with fibrinous base, hyperaemic periphery, slightly irregular mucosal surface; biopsy excluded neoplastic changes. After PPI and anti-HP therapy, the patient ultimately required surgical treatment with duodenotomy, antrectomy and Roux-en-Y gastrojejunostomy [Adapted from Open Access ref. no [18]]

**Fig. 12:**
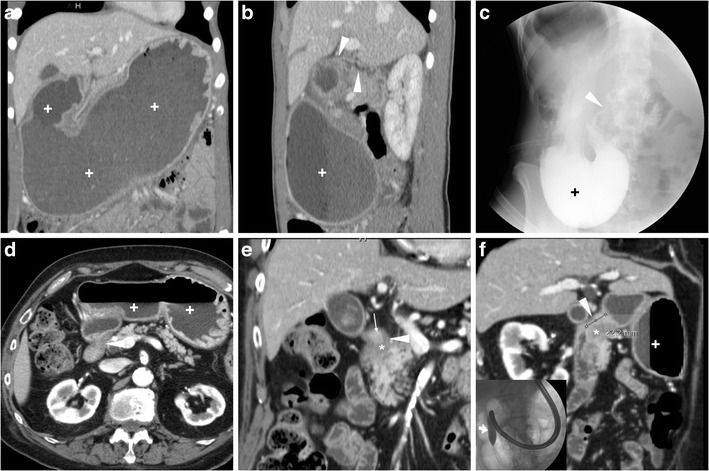
A young 21-year-old female with history of HP infection experienced gastric dilatation, relieved with nasogastric intubation. Despite inability to drink, contrast-enhanced CT (**a, b**) showed markedly dilated fluid-filled stomach (+), contracted duodenal bulb with 3-cm long stricture (arrowheads in b) without mural thickening, abnormal solid tissue and extrinsic masses. Upper digestive double-contrast fluoroscopy (**c**) showed dilated stomach with stagnant barium (+) and poorly distensible gastric outlet (arrowhead). Endoscopy confirmed deformed, impassable stricture and two small-sized pyloric ulcers with bioptic finding of severe acute and chronic inflammation. A 88-year-old diabetic male suffered from vomiting, epigastric pain and dark stools. Contrast-enhanced CT (**d…f**) confirmed radiographic finding of gastric dilatation unrelieved by nasogastric tube, with intraluminal stagnant fluid (+) and depicted a 2-cm pyloric-duodenal stricture (arrowheads in **e**, **f**) with subtle submucosal hypoattenuation (*) and mucosal hyperenhancement (thin arrow in **e**) but no signs of mass-forming or infiltrative disease. Biopsies confirmed post-inflammatory stricture, which was treated by endoscopic dilatation

Plain abdominal radiographs generally show gastric overdistension with scarce or no air distally (Fig. 11a). Before endoscopy and biopsy, CT imaging allows confirming mechanical nature, level (transition point) and probable cause of GOO. The stagnant gastric content provides luminal distension without administering oral contrast, which is poorly tolerated and may potentially cause inhalation. GOO secondary to PUD is suggested by more or less symmetric, oedematous pyloric-duodenal mural thickening with interrupted mucosal enhancement or ulcer outpouching (Fig. 11). Alternatively, in our experience it may present as a tight stricture measuring 2–3 cm in length, without abnormal mural thickening, solid tissue or extrinsic mass (Fig. 12). Endoscopic balloon dilatation of benign GOO is feasible but rarely allows long-term cure [5, 7, 19].

### Differential diagnosis of PUD-related gastric outlet obstruction

**Fig. 13:**
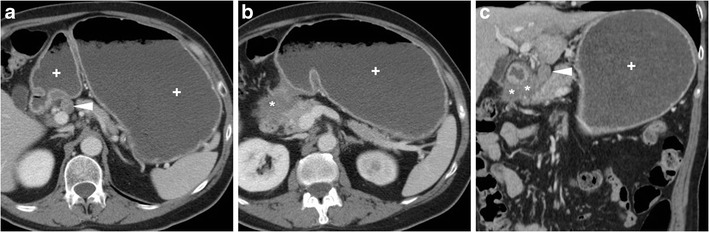
In a 77-year-old male, obstructing duodenal carcinoma appeared at CT as an eccentric, poorly enhancing tissue (*) abutting the proximal duodenum, causing upstream gastric fluid-filled dilatation (+). Note adjacent round-shaped solid lymphadenopathy (arrowheads)

**Fig. 14:**
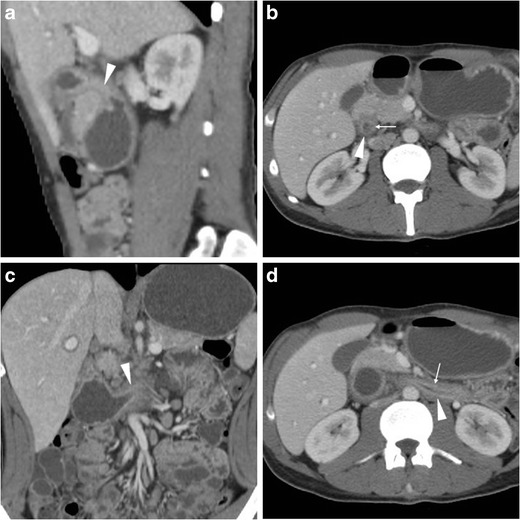
A 31-year-old male with history of ileocecal resection for Crohn’s disease (CD) seven years earlier underwent CT-enterography with oral polyethylene glycol solution: the endoscopically diagnosed impassable stricture was well depicted between first and second duodenum (arrowheads in **a**, **b**), and a second, longer cd lesion was seen the fourth duodenum (arrowheads in **c**, **d**). Both strictures showed characteristic mural stratification and enhancing mucosa (thin arrows) of active CD

**Fig. 15:**
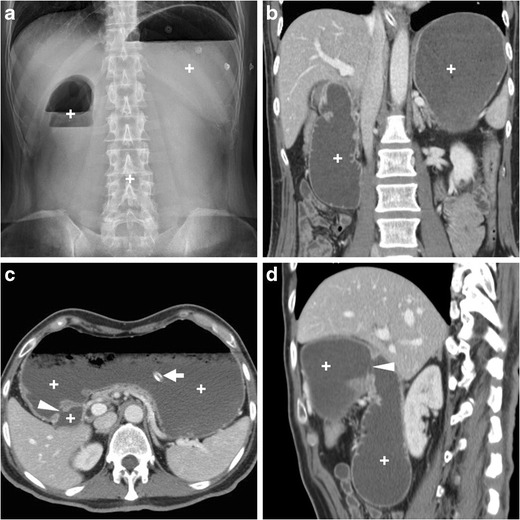
A 62-year-old diabetic male with recurrent metabolic decompensation and recent foot infection suffered from vomiting, abdominal pain and distension. With negative endoscopic findings, diabetic gastroparesis was diagnosed and treated with parenteral nutrition and peristaltic drugs

Compared to PUD, other causes of GOO are much more prevalent. Malignancies (Figs. 6, and 13) usually manifest with dysphagia, weight loss and anaemia, and should be systematically suspected in individuals over 50 years of age. In young people and adolescents, Crohn’s disease (CD, Fig. 14) should be considered, albeit rare in the upper digestive tract without typical ileocecal involvement [20]. Delayed gastric emptying or gastroparesis (Fig. 15) secondary to diabetes, central nervous system or smooth muscle disorders such as scleroderma and amyloidosis, post-viral syndromes and medications such as anticholinergics and narcotics may mimic GOO [11–13, 19, 20].

## Fistulisation

**Fig. 16:**
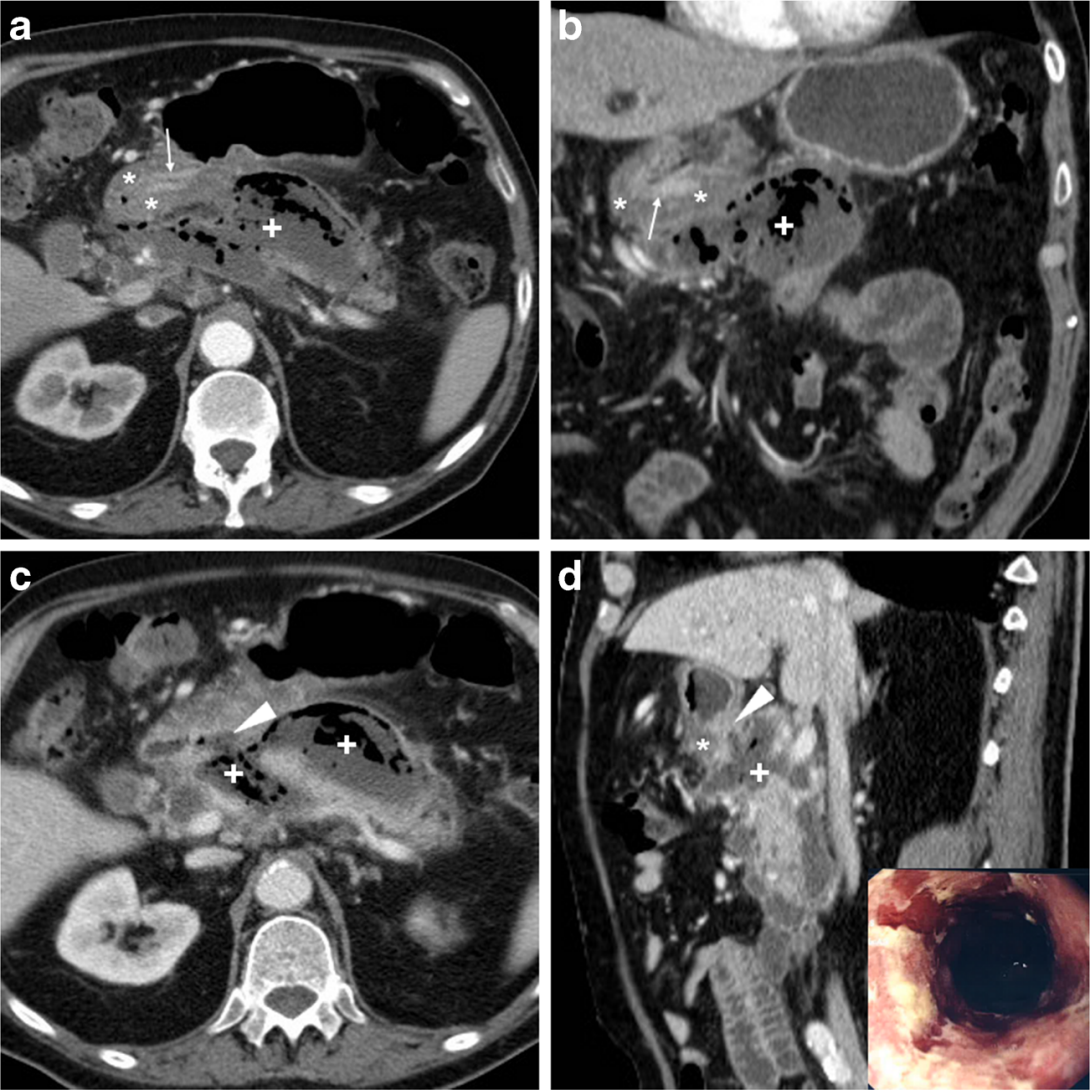
In an 84-year-old male with malaise, appetite loss, epigastric tenderness and protracted low-grade fever, contrast-enhanced CT detected a vast retroperitoneal abscess (+) with some internal gas and residual enhancing pancreatic parenchyma at body and tail, which was indissociable from the distal gastric antrum and pylorus: the latter showed circumferential mural thickening with oedematous submucosa (*) and hyperenhancing mucosa (thin arrows). Additionally, focal communication (arrowheads in **c**, **d**) was identified between pancreatic abscess and inflamed digestive tract. The fistulisation site corresponded to endoscopic finding (image in inset **d**) of large posterior bulbar ulcer. Laparotomic surgery required extensive dissection, confirmed a vast, stinky purulent and necrotic collection, fixed to the stomach and deformed pylorus, and was completed with gastric resection and Billroth-II gastrojejunostomy. Pathology confirmed severe transmural ulcerated peptic gastro-duodenitis, without malignant changes [Adapted from Open Access ref. no [21]]

**Fig. 17:**
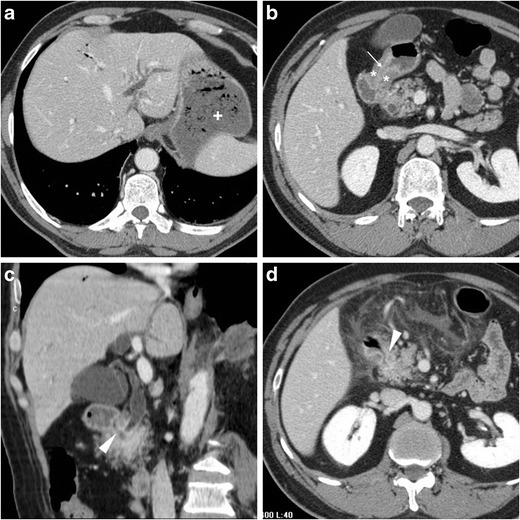
In a 53-year-old male with longstanding history of untreated duodenal ulcer, contrast-enhanced CT **(a...c)** revealed overdistended stomach (+) with fluid, normal-appearing gallbladder, minimal intrahepatic biliary dilatation in left liver lobe and some air in peripheral intrahepatic ducts (arrows) without previous surgery or biliary intervention. The antropyloric tract showed oedematous submucosa (*) and enhancing mucosa (thin arrow in **b**), and closely adhered to the ventral aspect of pancreatic head, and a thin fluid-containing track suggesting fistulisation (arrowhead in **c**) was identifiable between proximal duodenum and distal choledochus. Endoscopy showed duodenal bulb deformation from chronic PUD without active ulcers, and endoscopic retrograde cholangiopancreatography was unsuccessful. During a bout of acute pancreatitis, repeated CT (**d**) confirmed choledocho-duodenal fistula (arrowhead). Prolonged medical therapy ultimately allowed resolution of symptoms [Adapted with permission from ref. no [22]]

**Fig. 18:**
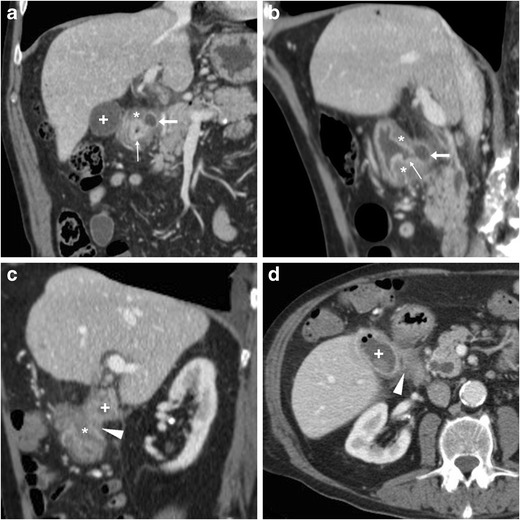
A 78-year-old male with history of chronic NSAID use had initial contrast-enhanced CT (**a, b**) finding of circumferential thickening of pylorus and proximal duodenum with oedematous submucosa (*), enhancing mucosa (thin arrows) and posterior ulcer outpouching (arrows); ventrally, the normal-appearing gallbladder (+) was in contact with the affected duodenal bulb. Fifteen months later, the patient experienced melaena and abdominal pain: repeated CT (**c, d**) showed development of communication (arrowheads) between thickened duodenum (*) and contracted gallbladder (+) with intraluminal air and some dependent sludge or blood

Contrary to intraperitoneal perforation, which causes free peritonitis in the abdominal cavity, posterior duodenal ulcers may occasionally penetrate into the retroperitoneum or fistulise to adjacent organs such as the pancreas (Fig. 16), common bile duct (Fig. 17), gallbladder (Fig. 18) or liver. Clinical manifestations of retroperitoneal infections may be either hyperacute (epigastric pain and tenderness, fever) or insidious (abdominal or back pain, malaise weight loss). Leukocytosis and elevation of acute phase reactants, serum amylase, lipase and hepatic transaminases are generally observed [23–27].

CT consistently shows peripherally enhancing pancreatic abscesses (Fig. 16), which most commonly result from superinfection of post-necrotic collections following severe acute pancreatitis. Conversely, the presence of air-attenuation components and signs of severe gastro-duodenitis suggest the correct diagnosis of fistulising PUD, which is crucial to prevent failed laparoscopy. Open surgical treatment including gastric resection or duodenectomy is generally required [23, 24].

Alternatively, PUD may fistulise into the gallbladder or biliary tract, causing superimposition of jaundice and ascending cholangitis on chronic PUD complaints. Spontaneous pneumobilia without previous surgery or instrumentation is the CT hallmark of bilio-digestive fistulas. Other suggestive findings include close adhesion and possible communication between posterior duodenum and either ventral aspect of pancreatic head (Fig. 17) or gallbladder (Fig. 18). The gold standard technique for biliary fistulas, endoscopic retrograde cholangiopancreatography (ERCP) is often unfeasible because of duodenal deformity and narrowing. Surgical management is indicated in cases refractory to medical therapy. Coledochoduodenal fistulas from PUD are very uncommon compared to cholelithiasis-related perforation into a normal duodenum: the latter are suggested by contracted gallbladder with mural thickening, and may give rise to further complications, such as gallstone ileus and Bouveret syndrome [7, 22, 25, 27, 28].

## Conclusion

Albeit not the diagnostic technique of choice for suspected PUD, multidetector CT may allow aware radiologists to diagnose both uncomplicated PUD and complications such as haemorrhage, GOO, and biliary or pancreatic fistulisation, thus allowing for timely appropriate treatment [5, 7, 19].
